# Immunoregulatory Interplay Between Arginine and Tryptophan Metabolism in Health and Disease

**DOI:** 10.3389/fimmu.2019.01565

**Published:** 2019-07-09

**Authors:** Giada Mondanelli, Alberta Iacono, Massimo Allegrucci, Paolo Puccetti, Ursula Grohmann

**Affiliations:** Department of Experimental Medicine, University of Perugia, Perugia, Italy

**Keywords:** arginase 1, indoleamine 2, 3 dioxygenase 1, polyamine, dendritc cells, autoimmunity, neoplasia

## Introduction

Over evolution, some amino-acid catabolic pathways have become critical checkpoints in immunity (1–3). The associated immunoregulatory effects rely on the depletion of specific amino acids in the microenvironment and/or generation of biologically active metabolites (4). Consumption of l-arginine (Arg) by arginase 1 (ARG1) represents a well-known immunoregulatory mechanism exploited by M2 macrophages (5) and myeloid-derived suppressor cells (MDSCs) (6–8) in tumor settings. ARG1 is also expressed by human neutrophils (9). Indoleamine 2,3-dioxygenase 1 (IDO1)—a powerful immunosuppressive enzyme catalyzing the first, rate limiting step in l-tryptophan (Trp) catabolism—depletes Trp and produces immunoregulatory molecules collectively known as kynurenines (10–13). High IDO1 expression and catalytic activity occur in dendritic cells (DCs)—professional antigen presenting cells—in response to interferon-γ (IFN-γ) (8, 10, 11). Unlike ARG1, IDO1 is also endowed with non-enzymatic signaling activity in DCs that, in the presence of transforming growth factor-β (TGF-β) in microenvironments, leads to durable immunoregulatory effects (14, 15). In conventional DCs (cDCs), a relay pathway—marked by the sequential activation of ARG1 and IDO1—promotes a potent immunoregulatory phenotype (8, 16, 17). In this setting, spermidine, i.e., a polyamine produced downstream of the ARG1-dependent pathway (18), is capable of triggering IDO1 phosphorylation and signaling, and thus may represent the critical molecular interconnection between the two enzymes (8, 16). Here, we discuss the possible protective vs. pathogenetic roles of the interplay between IDO1 and ARG1 in reprogramming immune cell functions in neoplasia and autoimmune diseases.

## The ARG1 and IDO1 Interplay as Physiologic Immune Checkpoint

As all biological processes, immune responses rely on both energy-consuming and energy-producing pathways (19). The availability of specific substances and the immunological signature of the microenvironment directly control immune cell fate and functions. Pathogen-associated molecular pattern (PAMPs) and damage-associated molecular pattern (DAMPs) molecules (recognized by pattern recognition receptors such as Toll-like receptors or TLRs) as well as amino acids, glucose, and fatty acids drive T-cell proliferation. Indeed, among immune cells, T lymphocytes are particularly dependent on nutrient availability and such feature (known as auxotrophy) has evolved as biological containment strategy that promotes the life-or-death decision (19).

By reducing the supply of indispensable amino acids, IDO1 and ARG1 directly suppress T cell proliferation and differentiation. The inadequacy of Arg and Trp substrates promotes a state of quiescence, whereby non-essential functions are temporarily quenched, including the cell cycle progression in the G_0_-G_1_ phase and the expression/activation of the TCR ζ-chain (2, 20, 21). IDO1 and ARG1 are indeed considered as physiological checkpoints ensuring a short-lived immunosuppression in normal pregnancies. In the placenta, DCs and extravillous trophoblasts highly expressing IDO1 and ARG1 secure a reversible T cell hyporesponsiveness and thus the survival of the fetus *in utero* (22, 23).

The activity of ARG1 and IDO1 translates not only into amino acid deprivation, but also in the production of metabolites endowed with several physiologic effects. l-kynurenine and spermidine, derived from Trp and Arg, respectively, are clear archetypes of non-inert byproducts that can influence immune and non-immune cell functions. In particular, l-kynurenine, by engagement of the aryl hydrocarbon receptor (AhR; a ligand-activated transcription factor), favors the differentiation of regulatory T (Treg) cells and induces IDO1 expression in DCs (24). On the other hand, the polycationic spermidine regulates cell growth and proliferation, and it affects several signal transducing pathways by interacting with ion channels, membrane receptors, and kinases (18).

Under specific conditions (as those dominated by TGF-β), Arg and Trp metabolic pathways are co-activated, thus potentiating the immunoregulatory phenotype of DCs and MDSCs (8, 25). The intimate relationship between ARG1 and IDO1 is allowed by spermidine, which activates the non-enzymatic functions of IDO1 and thus reprograms the cDC toward a long-term, immunoregulatory phenotype. More specifically, through Src kinase activation, spermidine induces the phosphorylation of IDO1, which, in turn, behaves as signaling molecule, promoting activation of the non-canonical pathway of NF-κB and induction of TGF-β1 and IDO1 expressions (3, 8). Contrary to spermidine, the small molecule nitric oxide (NO; derived from the Arg breakdown catalyzed by NO synthase) negatively regulates Trp metabolism, as it directly binds the heme prosthetic group and thus blunts the enzymatic function of IDO1 (26). However, besides this effect that would dampen IDO1-mediated immunosuppression, high levels of NO can combine with superoxide anion thus generating reactive nitrogen species that compromise both the activity and migration of T cells at the tumor site (27). Of note, it has been recently shown that AhR can sustain intracellular polyamines production at least in neoplastic conditions (28). However, whether such positive modulation belongs to a physiologic, bi-directional regulation program, where Trp metabolites and/or IDO1 itself affect ARG1 functions, has not been investigated yet.

## ARG1 and IDO1 in Neoplasia

Difference in the metabolism of normal and cancer cells underlie the quest for more specific and less toxic therapies than those currently used. Tumor development is conditioned by genetic changes in malignant cells, immunological tolerance, and immunosuppression (29). At the initial stages of carcinogenesis, the immune system is capable of anti-tumor activity; however, cancer progression compromises the action of T helper type 1 (Th1)/Th2/Th17 lymphocytes via Treg cells, tumor-associated macrophages (TAMs), and MDSCs, resulting in immunosuppression and loss of reactivity to tumor antigens (30, 31). Recently, much attention has been dedicated to the influence of Arg and Trp metabolic pathways on both tumor cell growth and host's immune antitumor response. Arg is essential for the maturation of the TCR ζ-chain, and its deprivation impairs T cell ability to activate tumor immunity. MDSCs deplete Arg because they express high levels of ARG1, and their number increases 4–10 times depending on the type of cancer. For these reasons, in cancer immunotherapy studies, the effects of both deprivation and supplementation of Arg have been tested, the former on the assumption that tumors may be Arg auxotrophic, and the latter in an effort to counteract the detrimental effect of ARG1-competent, tumor-associated MDSCs on the host antitumor response. Overall, seemingly contradictory results were found in such oncological therapies based on the deprivation or supplementation of Arg, and those results are not easily reconciled (29). In particular, the high efficacy of subtracting Arg to Arg auxotrophic tumors may hardly explain *per se* the global protective effect of this maneuver, in that most tumors may ultimately activate the arginine-succinate synthetase (ASS1) pathway that enables synthesis of Arg from citrulline. The recent finding of a supportive influence of ARG1 on IDO1-dependent tolerogenesis (8)—which would impair host antitumor responses—suggests that it is not the Arg subtraction to the tumor that matters so much as the impairment of ARG1's supportive role in allowing full expression of the IDO1 mechanism in suppressing antitumor responses. In fact, ARG1^+^ MDSCs, obtained by cell incubation with medium derived from mouse melanoma cells, can condition DCs to acquire an IDO1-dependent, immunoregulatory phenotype *in vivo* via production of polyamines (8, 16). Therefore, these data would sustain the existence of an immunosuppressive cross-talk mechanism between distinct cells present in tumor stroma and expressing ARG1 and/or IDO1 (32). The mechanisms whereby IDO1 acts as an immunosuppressant are multiple, and they are detailed elsewhere (2, 10, 11, 33).

There are, however, clinical settings where pharmacological administration of Arg resulted in cytoreductive effects in patients with Arg non-auxotrophic tumors (29). Paradoxical as it seems, this effect could again be explained by the relationship between ARG1 and IDO1 in immune cells. Increased ARG1 activity might lead to IDO1-dependent Trp starvation in cancer cells. Because Trp is an essential and the rarest of all amino acids, this likely results in an overall proteostatic action that affects fast-growing tumors, as discussed elsewhere in detail (3).

With specific regard to Arg auxotrophy, this phenomenon takes place in certain tumors and is caused by the silencing of ASS1 or arginine lyase genes. Those tumors are characterized by an intrinsic chemoresistance and thus a poor prognosis. Nevertheless, on a positive note, Arg auxotrophy theoretically favors the treatment of these tumors with Arg-degrading enzymes. Among the most frequently applied Arg-degrading agents are arginine deiminases (ADI) from bacteria. The antitumor effects of ADI derived from different bacteria have been extensively studied *in vitro* and *in vivo* [for review, see (34)]. Mycoplasma-derived ADI-PEG20 is the one most commonly used and is under clinical investigation as a single agent therapeutic as well as in combination with other chemotherapeutic compounds. Mechanistically, ADI reduces metabolic activity in tumor cells, contributing to autophagy, senescence, and apoptosis in Arg auxotrophic cells (34). Although clinical trials are promising, the development of resistance after initial treatment is challenging, as illustrated above. Furthermore, an ADI interference within the tumor microenvironment is to be considered. Again, non-specific subtraction of the substrate for ARG1 may indirectly affect the host response to the tumor via effects on IDO1.

Another important issue in cancer is the expression of Arg and Trp transporters in tumor and immune cells. Among Arg carriers (cationic amino acid transporters or CATs), the most important appear to be CAT1, which is constitutively expressed in several tissues, and CAT2B, normally inducible by inflammatory cytokines (35). CAT1 is often overexpressed by tumor cells, and event that can favor tumor growth. In an experimental model of prostate cancer, CAT2B, which allows a rapid transport of Arg into the cell, is expressed at higher levels in tumor-infiltrating as compared to peripheral MDSCs (36). Moreover, the upregulation of CAT2 is coordinated with the induction of both NOS2 and ARG1, thus further favoring Arg uptake by MDSCs at the tumor site. Subsets of human melanoma cells are also characterized by very high levels of CAT2B expression, possibly due to the secretion of inflammatory mediators by the tumor cells themselves (35). Overexpression of Trp carriers (mainly, LAT1/CD98 and SLC6A14) is also involved in the increased proliferation and chemoresistance of several tumor cell types (37). Because SLC6A14 is a broad specific amino acid transporter that can also transfer Arg and its expression can be upregulated by IDO1 (by a mechanism not identified yet) (38), the “doors” for the cell entrance of Arg and Trp may represent suitable cancer drug targets capable of interfering with both ARG1 and IDO1 pathways (39).

Therefore, new insight is definitely needed into the molecular mechanisms underlying the antitumor effects of Arg starvation in both host and tumor, which might facilitate the refinement of IDO1 inhibitory approaches in cancer immunotherapy.

## ARG1 and IDO1 in Autoimmunity

The use of checkpoint inhibitors in tumor immunotherapy is frequently accompanied by the development of autoimmune diseases (40), suggesting that the exploitation of immune checkpoint molecules could be a valid therapeutic means in autoimmunity (4, 41). Because both ARG1 and IDO1 act as immune checkpoint mechanisms in neoplasia, their functional “alliance” in specific immune cells could be remarkably effective in controlling adaptive immunity toward auto-antigens.

IDO1 is defective in DCs of non-obese diabetic (NOD) mice (42), an experimental model of human autoimmune diabetes (type 1 diabetes or T1D), and maneuvers aimed at enhancing its expression and activity will exert therapeutic effects in prediabetic and also overtly diabetic animals (43, 44). In T1D patients, a significantly reduced IDO1 expression can be observed in peripheral blood mononuclear cells (PBMCs) (17) and in pancreatic β cells (45), normally producing insulin. In PBMCs, the defect can be corrected by tocilizumab, a blocker of the interleukin 6 (IL-6) receptor, which inhibits the IL-6–dependent, IDO1 proteasomal degradation (17). In T1D, although its expression and function in immune cells remains unclear, endothelial ARG1 induces the vascular dysfunction associated with hyperglycemia (46). Moreover, administration of difluoromethylornithine (DFMO), a potent inhibitor of polyamine production, protects NOD mice from the development of diabetes.

In experimental models of rheumatoid arthritis (RA), an inflammatory/autoimmune disease of the capsule surrounding joints, lack of IDO1 expression reduces the time to develop a more severe disease (47). Moreover, the protective effects of interferon-α rely on the activation of a TGF-β/IDO1 axis in plasmacytoid DCs (48). Although ARG1^+^ M2 macrophages contribute to resolve arthritis inflammation in mice (49), ARG1 activity may be responsible for subclinical endothelial dysfunction also in RA patients (50). Interestingly, methotrexate, an immunosuppressive drug widely used in RA, greatly inhibits the synthesis of polyamines in lymphocytes of RA patients (51).

A definitely clearer picture is emerging in autoimmune neuroinflammation. Administration of 3-hydroxyanthranilic acid (3-HAA; a Trp metabolite of the kynurenine pathway) (52) or of an orally active synthetic derivative thereof (53) ameliorates neuroinflammation and paralysis in mice with acute experimental autoimmune encephalomyelitis (EAE), a model for multiple sclerosis (MS). Moreover, 3-HAA–treated DCs express higher levels of TGF-β and induce the generation of Treg cells (52). Conversely, administration of 1-methyltryptophan (1-MT), a standard inhibitor of IDO1, exacerbates the clinical course of EAE (54, 55). In leukocytes infiltrating the spinal cord of untreated mice, IDO1-expressing cells exhibit the same morphology as activated macrophages/microglia (54). VCE-004.8, a semisynthetic cannabinoid, protects from EAE, possibly by upregulating ARG1 in macrophages and microglia (56). A Lewis^X^ trisaccharide of schistosome eggs reduces EAE severity by a TLR-mediated mechanism that enhances both ARG1 and IDO1 expression in CD11b^+^Ly-6C^hi^ inflammatory monocytes (57). Expression and activity of ARG1 and IDO1 are significantly reduced in PBMCs from MS patients as compared to healthy control subjects (58). Spermidine, the polyamine produced downstream ARG1, protects from autoimmune-directed demyelination of neurons in acute EAE (59). The effect appears to be related to an immunosuppressive function acquired by ARG1^+^ macrophages, since (i) their depletion or the administration of an ARG1 inhibitor abolishes spermidine therapeutic activity *in vivo* and (ii) the polyamine induces ARG1 in macrophages (59).

Therefore, although in both T1D and RA the pathways of Arg and Trp metabolism do not seem to be properly interlinked (and this may require cautions when attempting immunotherapies potentiating both ARG1 and IDO1), in MS, the pieces of evidence, when put together, would suggest that the induction of the immunosuppressive interplay between ARG1 and IDO1 would represent a valid therapeutic objective.

## Conclusions and Perspectives

**Figure 1:**
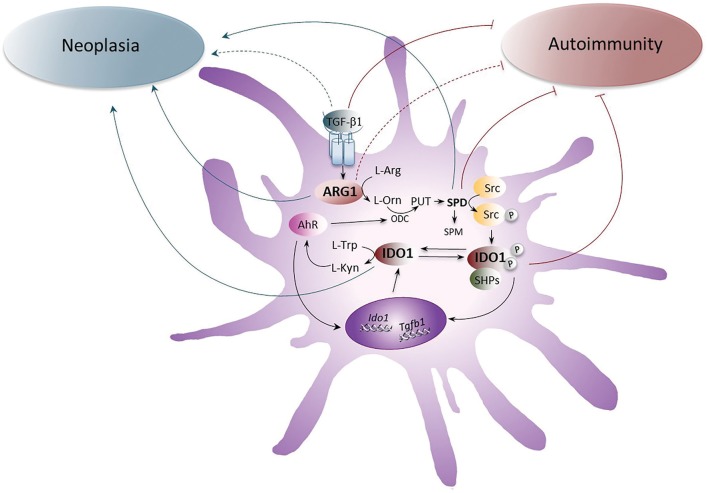
The role of ARG1 and IDO1 in neoplasia and autoimmunity. The up-regulation of ARG1 activity, induced by the cytokine TGF-β, transforms l-arginine (l-Arg) into l-ornithine (l-Orn), which is further metabolized by ornithine decarboxylase (ODC) into polyamines (PUT, putrescine; SPD, spermidine; and SPM, spermine). SPD, through the activation of the Src kinase, promotes the phosphorylation of IDO1 and thus favors the initiation of immunoregulatory signaling events in DCs. Once phosphorylated, IDO1 recruits tyrosine phosphatases (SHPs) and promotes a signaling pathway that upregulates the expression of genes coding for IDO1 and TGF-β, thus creating a self-sustaining circuitry responsible for the maintenance of immune tolerance over the long-term. Moreover, IDO1 catalyzes the conversion of l-tryptophan (l-Trp) into l-kynurenine (l-Kyn), which activates the aryl hydrocarbon receptor (AhR). AhR further induces IDO1 expression in DCs and sustains the production of polyamines by up-regulating ODC. Whereas the pathogenetic and protective role of TGF-β, SPD, and IDO1 in neoplasia and autoimmunity, respectively, have been demonstrated, the role of ARG1 has been unclear and would require further investigations. Gray arrows indicate the pathogenetic effects of IDO1, ARG1, SPD, and TGF-β1 receptor signaling in neoplasia and brown arrows indicate the putative protective effects of IDO1, SPD, ARG1, and TGF-β1 receptor signaling in autoimmune diseases. Dotted lines are for molecules whose role is still unclear.

In neoplasia, both ARG1 and IDO1 are often overexpressed, either singly in tumor cells themselves (IDO1) or in association (i.e., both enzymes) in MDSCs and DCs, and they contribute to the impairment of the host anti-tumor immunity. However, the effect of Arg starvation on tumor cells may dampen their proliferation and therefore ARG1 inhibition as therapeutic strategy may have some caveats (Figure 1). In the majority of autoimmune disorders, the bulk of data would suggest that IDO1, expressed by either DCs or macrophages, stands out as an effective immune checkpoint molecule. In contrast, more often than not, ARG1 appears to be more pathogenetic than protective, possibly owing to the enzyme capacity to subtract Arg for NO production, which can be necessary for the resolution of damages induced by autoimmunity (4, 60). However, in autoimmune neuroinflammation, the available cues would indicate that both ARG1 and IDO1, expressed by macrophages and/or DCs, act as immune checkpoint molecules in EAE and that spermidine, i.e., the molecular connection between the two enzymes in a physiologic setting (8), exerts significant therapeutic effects on its own. Therefore, further investigations on Arg metabolism in neoplasia and autoimmune disorders and its possible cross-talk with IDO1 are needed for a full understanding of its role, protective vs. pathogenetic.

## Author Contributions

GM, AI, MA, PP, and UG equally contributed in writing the manuscript. UG supervised the final form.

### Conflict of Interest Statement

The authors declare that the research was conducted in the absence of any commercial or financial relationships that could be construed as a potential conflict of interest.

## References

[1] Murray PJ. Amino acid auxotrophy as a system of immunological control nodes. Nat Immunol. (2016) 17:132–9. 10.1038/ni.3323 26784254 PMC4893777

[2] Grohmann U Bronte V. Control of immune response by amino acid metabolism. Immunol Rev. (2010) 236:243–64. 10.1111/j.1600-065X.2010.00915.x 20636821

[3] Grohmann U Mondanelli G Belladonna ML Orabona C Pallotta MT Iacono A. Amino-acid sensing and degrading pathways in immune regulation. Cytokine Growth Factor Rev. (2017) 35:37–45. 10.1016/j.cytogfr.2017.05.004 28545736

[4] Mondanelli G Iacono A Carvalho A Orabona C Volpi C Pallotta MT. Amino acid metabolism as drug target in autoimmune diseases. Autoimmun Rev. (2019) 18:334–8. 10.1016/j.autrev.2019.02.004 30797943

[5] Sica A Mantovani A. Macrophage plasticity and polarization: *in vivo* veritas. J Clin Investig. (2012) 122:787–95. 10.1172/JCI59643 22378047 PMC3287223

[6] Gabrilovich DI Nagaraj S. Myeloid-derived suppressor cells as regulators of the immune system. Nat Rev Immunol. (2009) 9:162–74. 10.1038/nri2506 19197294 PMC2828349

[7] Marigo I Dolcetti L Serafini P Zanovello P Bronte V. Tumor-induced tolerance and immune suppression by myeloid derived suppressor cells. Immunol Rev. (2008) 222:162–79. 10.1111/j.1600-065X.2008.00602.x 18364001

[8] Mondanelli G Bianchi R Pallotta MT Orabona C Albini E Iacono A. A relay pathway between arginine and tryptophan metabolism confers immunosuppressive properties on dendritic cells. Immunity. (2017) 46:233–44. 10.1016/j.immuni.2017.01.005 28214225 PMC5337620

[9] Munder M. Arginase: an emerging key player in the mammalian immune system. Br J Pharmacol. (2009) 158:638–51. 10.1111/j.1476-5381.2009.00291.x 19764983 PMC2765586

[10] Grohmann U Fallarino F Puccetti P. Tolerance, DCs and tryptophan: much ado about IDO. Trends Immunol. (2003) 24:242–8. 10.1016/S1471-4906(03)00072-3 12738417

[11] Mellor AL Munn DH. IDO expression by dendritic cells: tolerance and tryptophan catabolism. Nat Rev Immunol. (2004) 4:762–74. 10.1038/nri1457 15459668

[12] Puccetti P Grohmann U. IDO and regulatory T cells: a role for reverse signalling and non-canonical NF-kappaB activation. Nat Rev Immunol. (2007) 7:817–23. 10.1038/nri2163 17767193

[13] Wang J McGuire TR Britton HC Schwarz JK Loberiza FR Meza JL. Lenalidomide and cyclophosphamide immunoregulation in patients with metastatic, castration-resistant prostate cancer. Clin Exp Metastasis. (2015) 32:111–24. 10.1007/s10585-015-9696-3 25617965

[14] Pallotta MT Orabona C Volpi C Vacca C Belladonna ML Bianchi R. Indoleamine 2,3-dioxygenase is a signaling protein in long-term tolerance by dendritic cells. Nat Immunol. (2011) 12:870–8. 10.1038/ni.2077 21804557

[15] Mondanelli G Volpi C. Differentiation of myeloid-derived suppressor cells from murine bone marrow and their co-culture with splenic dendritic cells. Bio Protocol. (2017) 7:e2558. 10.21769/BioProtoc.2558 29130057 PMC5681245

[16] Mondanelli G Ugel S Grohmann U Bronte V. The immune regulation in cancer by the amino acid metabolizing enzymes ARG and IDO. Curr Opin Pharmacol. (2017) 35:30–9. 10.1016/j.coph.2017.05.002 28554057

[17] Orabona C Mondanelli G Pallotta MT Carvalho A Albini E Fallarino F. Deficiency of immunoregulatory indoleamine 2,3-dioxygenase 1in juvenile diabetes. JCI Insight. (2018) 3:96244. 10.1172/jci.insight.96244 29563329 PMC5926942

[18] Madeo F Eisenberg T Pietrocola F Kroemer G. Spermidine in health and disease. Science. (2018) 359: eaan2788. 10.1126/science.aan2788 29371440

[19] Wang A Luan HH Medzhitov R. An evolutionary perspective on immunometabolism. Science. (2019) 363:eaar3932. 10.1126/science.aar3932 30630899 PMC6892590

[20] Geiger R Rieckmann JC Wolf T Basso C Feng Y Fuhrer T. L-Arginine modulates T cell metabolism and enhances survival and anti-tumor activity. Cell. (2016) 167:829–42.e13. 10.1016/j.cell.2016.09.031 27745970 PMC5075284

[21] Fallarino F Grohmann U You S McGrath BC Cavener DR Vacca C. The combined effects of tryptophan starvation and tryptophan catabolites down-regulate T cell receptor zeta-chain and induce a regulatory phenotype in naive T cells. J Immunol. (2006) 176:6752–61. 10.4049/jimmunol.176.11.6752 16709834

[22] Munn DH Zhou M Attwood JT Bondarev I Conway SJ Marshall B. Prevention of allogeneic fetal rejection by tryptophan catabolism. Science. (1998) 281:1191–3. 10.1126/science.281.5380.1191 9712583

[23] Kropf P Baud D Marshall SE Munder M Mosley A Fuentes JM. Arginase activity mediates reversible T cell hyporesponsiveness in human pregnancy. Eur J Immunol. (2007) 37:935–45. 10.1002/eji.200636542 17330821 PMC2699382

[24] Gutiérrez-Vázquez C Quintana FJ. Regulation of the immune response by the Aryl hydrocarbon receptor. Immunity. (2018) 48:19–33. 10.1016/j.immuni.2017.12.012 29343438 PMC5777317

[25] Yu J Du W Yan F Wang Y Li H Cao S Myeloid-derived suppressor cells suppress antitumor immune responses through IDO expression and correlate with lymph node metastasis in patients with breast cancer. J Immunol. (2013) 190:3783–97. 10.4049/jimmunol.1201449 23440412

[26] Thomas SR Mohr D Stocker R. Nitric oxide inhibits indoleamine 2,3-dioxygenase activity in interferon-gamma primed mononuclear phagocytes. J Biol Chem. (1994) 269:14457–64. 7514170

[27] De Sanctis F Sandri S Ferrarini G Pagliarello I Sartoris S Ugel S. The emerging immunological role of post-translational modifications by reactive nitrogen species in cancer microenvironment. Front Immunol. (2014) 5:69. 10.3389/fimmu.2014.00069 24605112 PMC3932549

[28] Bianchi-Smiraglia A Bagati A Fink EE Affronti HC Lipchick BC Moparthy S. Inhibition of the aryl hydrocarbon receptor/polyamine biosynthesis axis suppresses multiple myeloma. J Clin Investig. (2018) 128:4682–96. 10.1172/JCI70712 30198908 PMC6159960

[29] Szefel J Danielak A Kruszewski WJ. Metabolic pathways of L-arginine and therapeutic consequences in tumors. Adv Med Sci. (2018) 64:104–10. 10.1016/j.advms.2018.08.018 30605863

[30] Yu YR Ho PC. Sculpting tumor microenvironment with immune system: from immunometabolism to immunoediting. Clin Exp Immunol. (2019). 10.1111/cei.13293. [Epub ahead of print]. 30873592 PMC6642881

[31] Schreiber RD Old LJ Smyth MJ. Cancer immunoediting: integrating immunity's roles in cancer suppression and promotion. Science. (2011) 331:1565–70. 10.1126/science.1203486 21436444

[32] Ostrand-Rosenberg S Sinha P Beury DW Clements VK. Cross-talk between myeloid-derived suppressor cells (MDSC), macrophages, and dendritic cells enhances tumor-induced immune suppression. Sem Cancer Biol. (2012) 22:275–81. 10.1016/j.semcancer.2012.01.011 22313874 PMC3701942

[33] Orabona C Pallotta MT Grohmann U. Different partners, opposite outcomes: a new perspective of the immunobiology of indoleamine 2,3-dioxygenase. Mol Med. (2012) 18:834–42. 10.2119/molmed.2012.00029 22481272 PMC3409287

[34] Riess C Shokraie F Classen CF Kreikemeyer B Fiedler T Junghanss C. Arginine-depleting enzymes - an increasingly recognized treatment strategy for therapy-refractory malignancies. Cell Physiol Biochem. (2018) 51:854–70. 10.1159/000495382 30466103

[35] Kim SH Roszik J Grimm EA Ekmekcioglu S. Impact of l-arginine metabolism on immune response and anticancer immunotherapy. Front Oncol. (2018) 8:67. 10.3389/fonc.2018.00067 29616189 PMC5864849

[36] Cimen Bozkus C Elzey BD Crist SA Ellies LG Ratliff TL. Expression of cationic amino acid transporter 2 is required for myeloid-derived suppressor cell-mediated control of T cell immunity. J Immunol. (2015) 195:5237–50. 10.4049/jimmunol.1500959 26491198 PMC4655170

[37] Bhutia YD Babu E Ramachandran S Ganapathy V. Amino acid transporters in cancer and their relevance to “glutamine addiction”: novel targets for the design of a new class of anticancer drugs. Cancer Res. (2015) 75:1782–8. 10.1158/0008-5472.CAN-14-3745 25855379

[38] McCracken AN Edinger AL. Targeting cancer metabolism at the plasma membrane by limiting amino acid access through SLC6A14. Biochem J. (2015) 470:e17–9. 10.1042/BJ20150721 26341486 PMC4613721

[39] Badawy AA. Targeting tryptophan availability to tumors: the answer to immune escape? Immunol Cell Biol. (2018) 96:1026–34. 10.1111/imcb.12168 29888434

[40] Byun DJ Wolchok JD Rosenberg LM Girotra M. Cancer immunotherapy - immune checkpoint blockade and associated endocrinopathies. Nat Rev Endocrinol. (2017) 13:195–207. 10.1038/nrendo.2016.205 28106152 PMC5629093

[41] Orabona C Mondanelli G Puccetti P Grohmann U. Immune checkpoint molecules, personalized immunotherapy, and autoimmune diabetes. Trends Mol Med. (2018) 24:931–41. 10.1016/j.molmed.2018.08.005 30236470

[42] Grohmann U Fallarino F Bianchi R Orabona C Vacca C Fioretti MC. A defect in tryptophan catabolism impairs tolerance in nonobese diabetic mice. J Exp Med. (2003) 198:153–60. 10.1084/jem.20030633 12835483 PMC2196078

[43] Pallotta MT Orabona C Bianchi R Vacca C Fallarino F Belladonna ML. Forced IDO1 expression in dendritic cells restores immunoregulatory signalling in autoimmune diabetes. J Cell Mol Med. (2014) 18:2082–91. 10.1111/jcmm.12360 25215657 PMC4193887

[44] Mondanelli G Albini E Pallotta MT Volpi C Chatenoud L Kuhn C. The proteasome inhibitor bortezomib controls indoleamine 2,3-dioxygenase 1 breakdown and restores immune regulation in autoimmune diabetes. Front Immunol. (2017) 8:428. 10.3389/fimmu.2017.00428 28450863 PMC5390013

[45] Anquetil F Mondanelli G Gonzalez N Rodriguez Calvo T Zapardiel Gonzalo J Krogvold L. Loss of IDO1 expression from human pancreatic beta-cells precedes their destruction during the development of type 1 diabetes. Diabetes. (2018) 67:1858–66. 10.2337/db17-1281 29945890 PMC6110313

[46] Pernow J Jung C. The emerging role of arginase in endothelial dysfunction in diabetes. Curr Vasc Pharmacol. (2016) 14:155–62. 10.2174/1570161114666151202205617 26638796

[47] Criado G Simelyte E Inglis JJ Essex D Williams RO. Indoleamine 2,3 dioxygenase-mediated tryptophan catabolism regulates accumulation of Th1/Th17 cells in the joint in collagen-induced arthritis. Arthr Rheumatism. (2009) 60:1342–51. 10.1002/art.24446 19404944

[48] Chalise JP Pallotta MT Narendra SC Carlsson B Iacono A Namale J. IDO1 and TGF-beta Mediate protective effects of IFN-alpha in antigen-induced arthritis. J Immunol. (2016) 197:3142–51. 10.4049/jimmunol.1502125 27647832 PMC5055200

[49] Chen Z Andreev D Oeser K Krljanac B Hueber A Kleyer A. Th2 and eosinophil responses suppress inflammatory arthritis. Nat Commun. (2016) 7:11596. 10.1038/ncomms11596 27273006 PMC4899615

[50] Chandrasekharan UM Wang Z Wu Y Wilson Tang WH Hazen SL Wang S. Elevated levels of plasma symmetric dimethylarginine and increased arginase activity as potential indicators of cardiovascular comorbidity in rheumatoid arthritis. Arthr Res Ther. (2018) 20:123. 10.1186/s13075-018-1616-x 29884228 PMC5994036

[51] Nesher G Osborn TG Moore TL. *In vitro* effects of methotrexate on polyamine levels in lymphocytes from rheumatoid arthritis patients. Clin Exp Rheumatol. (1996) 14:395–9. 8871838

[52] Yan Y Zhang GX Gran B Fallarino F Yu S Li H. IDO upregulates regulatory T cells via tryptophan catabolite and suppresses encephalitogenic T cell responses in experimental autoimmune encephalomyelitis. J Immunol. (2010) 185:5953–61. 10.4049/jimmunol.1001628 20944000 PMC2998795

[53] Platten M Ho PP Youssef S Fontoura P Garren H Hur EM. Treatment of autoimmune neuroinflammation with a synthetic tryptophan metabolite. Science. (2005) 310:850–5. 10.1126/science.1117634 16272121

[54] Kwidzinski E Bunse J Aktas O Richter D Mutlu L Zipp F. Indolamine 2,3-dioxygenase is expressed in the CNS and down-regulates autoimmune inflammation. FASEB J. (2005) 19:1347–9. 10.1096/fj.04-3228fje 15939737

[55] Sakurai K Zou JP Tschetter JR Ward JM Shearer GM. Effect of indoleamine 2,3-dioxygenase on induction of experimental autoimmune encephalomyelitis. J Neuroimmunol. (2002) 129:186–96. 10.1016/S0165-5728(02)00176-5 12161035

[56] Navarrete C Carrillo-Salinas F Palomares B Mecha M Jiménez-Jiménez C Mestre L. Hypoxia mimetic activity of VCE-004.8, a cannabidiol quinone derivative: implications for multiple sclerosis therapy. J Neuroinflamm. (2018) 15:64. 10.1186/s12974-018-1103-y 29495967 PMC5831753

[57] Zhu B Trikudanathan S Zozulya AL Sandoval-Garcia C Kennedy JK Atochina O. Immune modulation by Lacto-N-fucopentaose III in experimental autoimmune encephalomyelitis. Clin Immunol. (2012) 142:351–61. 10.1016/j.clim.2011.12.006 22264636 PMC3288504

[58] Negrotto L Correale J. Amino acid catabolism in multiple sclerosis affects immune homeostasis. J Immunol. (2017) 198:1900–9. 10.4049/jimmunol.1601139 28130499

[59] Yang Q Zheng C Cao J Cao G Shou P Lin L. Spermidine alleviates experimental autoimmune encephalomyelitis through inducing inhibitory macrophages. Cell Death Different. (2016) 23:1850–61. 10.1038/cdd.2016.71 27447115 PMC5071574

[60] Krischel V Bruch-Gerharz D Suschek C Kröncke KD Ruzicka T Kolb-Bachofen V Biphasic effect of exogenous nitric oxide on proliferation and differentiation in skin derived keratinocytes but not fibroblasts. J Investig Dermatol. (1998) 111:286–91. 10.1046/j.1523-1747.1998.00268.x 9699731

